# Neurodegeneration in frontotemporal lobar degeneration and motor neurone disease associated with expansions in *C9orf72* is linked to TDP‐43 pathology and not associated with aggregated forms of dipeptide repeat proteins

**DOI:** 10.1111/nan.12292

**Published:** 2015-12-07

**Authors:** Y. Davidson, A. C. Robinson, X. Liu, D. Wu, C. Troakes, S. Rollinson, M. Masuda‐Suzukake, G. Suzuki, T. Nonaka, J. Shi, J. Tian, H. Hamdalla, J. Ealing, A. Richardson, M. Jones, S. Pickering‐Brown, J. S. Snowden, M. Hasegawa, D. M. A. Mann

**Affiliations:** ^1^ Clinical and Cognitive Sciences Research GroupInstitute of Brain, Behaviour and Mental HealthFaculty of Medical and Human SciencesUniversity of ManchesterSalford Royal HospitalSalfordUK; ^2^ Beijing University of Chinese MedicineDongzhimen HospitalBeijingChina; ^3^ London Neurodegenerative Diseases Brain BankDepartment of Basic and Clinical NeuroscienceInstitute of Psychiatry, Psychology and NeuroscienceKing's College LondonLondonUK; ^4^ Clinical and Cognitive Sciences Research GroupInstitute of Brain, Behaviour and Mental HealthFaculty of Medical and Human SciencesUniversity of ManchesterManchesterUK; ^5^ Department of Neuropathology and Cell BiologyTokyo Metropolitan Institute of Medical ScienceTokyoJapan; ^6^ Manchester MND Care CentreSalford Royal HospitalManchesterUK; ^7^ Cerebral Function UnitSalford Royal HospitalManchesterUK

**Keywords:** C9orf72, dipeptide repeat proteins, frontotemporal lobar degeneration, motor neurone disease, TDP‐43

## Abstract

**Aims:**

A hexanucleotide expansion in *C9orf72* is the major genetic cause of inherited behavioural variant Frontotemporal dementia (bvFTD) and motor neurone disease (MND), although the pathological mechanism(s) underlying disease remains uncertain.

**Methods:**

Using antibodies to poly‐GA, poly‐GP, poly‐GR, poly‐AP and poly‐PR proteins, we examined sections of cerebral cortex, hippocampus, thalamus, cerebellum and spinal cord, from 20 patients with bvFTD and/or MND bearing an expansion in *C9orf72* for aggregated deposits of dipeptide repeat proteins (DPR).

**Results:**

Antibodies to poly‐GA, poly‐GP and poly‐GR detected numerous rounded cytoplasmic inclusions (NCI) within granule cells of hippocampal dentate gyrus and those of the cerebellum, as well as ‘star‐burst’ shaped NCI in pyramidal neurones of CA3/4 region of hippocampus. NCI were uncommon in Purkinje cells, and only very rarely seen in anterior horn cells. Poly‐PA antibody detected occasional NCI within CA3/4 neurones alone, whereas poly‐PR antibody did not identify any NCI but immunostained the nucleus of anterior horn cells, CA3/4 neurones and Purkinje cells, in patients with or without expansion in *C9orf72*, as well as in normal controls. Poly‐GA antibody generally detected more DPR than poly‐GP, which in turn was greater than poly‐GR. All patients with bvFTD + MND or MND showed plentiful p62/TDP‐43 positive inclusions in remaining anterior horn cells.

**Conclusion:**

Degeneration and loss of anterior horn cells associated with expansions in *C9orf72* occurs in the absence of DPR, and implies that changes involving loss of nuclear staining for and a cytoplasmic aggregation of TDP‐43 are more likely to be the cause of this.

## Introduction

The major genetic cause of familial frontotemporal lobar degeneration (FTLD), and of familial motor neurone disease (MND), is associated with the possession of a hexanucleotide expansion in *C9orf72* gene, this occurring in about 20% cases of familial FTLD and 80% cases of familial MND 1, 2. The discovery of this genetic change has spawned a wealth of new knowledge and observation, although the exact pathological mechanism(s) underlying the expansion in *C9orf72* remains uncertain. A loss of function effect (haploinsufficiency) consequent upon a reduced output of C9orf72 protein has been suggested 2, 3, with the extent of the loss being dependent upon the degree of DNA methylation 4, 5, 6. Alternatively, the formation of both sense and antisense nuclear RNA foci has been demonstrated, both in human disease 2, 7, 8, 9 and in fly models 7. These might sequester RNA transcripts 2, 7, or other endogenous RNA binding proteins 8, 9, thereby interfering with the transcriptome. Lastly, a non‐ATG mediated (RAN) sense and antisense translation of the expansion itself leads to formation and cellular (usually cytoplasmic) accumulation of the dipeptide repeat proteins (DPR), poly‐GA, poly‐GR, poly‐GP, poly‐PA and poly‐PR, of presumed variable length 10, 11, 12, 13, 14, any, or all, of which might confer neurotoxicity. None of these three possible mechanisms are likely to be mutually exclusive, and in reality all could play some part in disease pathogenesis at different levels. Nevertheless, how any of these potential effects might translate into the TDP‐43 proteinopathy that characterises both conditions remains to be established.

With regard to DPR toxicity, it is still uncertain as to whether, or even which, particular DPR species can induce toxicity. May and colleagues 15, 16 showed that expression of poly‐GA containing DPR induced apoptosis and inhibited dendritic arborization in cultures of primary neurons. These authors noted that the poly‐GA aggregates formed in cells contained the transport factor Unc119 as a major co‐binding protein, and that expression of poly‐GA in neurons resulted in loss of Unc119 15, 16. Similar to poly‐GA, knockdown of Unc119 inhibited dendritic arborization and induced neurotoxicity, whereas overexpression of Unc119 partially rescued poly‐GA toxicity 15. Elsewhere, Zhang and co‐workers 17, observed that expression of poly‐GA in cultured cells and primary neurons lead to accumulation of aggregates, and caspase‐3 activation, impaired neurite outgrowth, proteasomal inhibition and ER stress. Against this, Mizielinska and colleagues reported that expression of arginine‐rich poly‐GR and poly‐PR proteins caused eye neurodegeneration in a *Drosophila* model of FTLD, whereas non‐arginine containing DPR (poly‐GA and poly‐PA) had no effect 18. These authors considered that the neurodegeneration was driven solely by expression of DPR, as the expression of RNA‐only repeats had no neurodegenerative effects despite the formation of RNA foci in both this and the DPR‐expressing constructs, and concluded that the expression of arginine‐rich DPR was the mediator of neurodegeneration and that RNA foci were of lesser importance 18. Similarly, Wen and colleagues, employing primary cortical and motor neurone cultures, live cell imaging and fly modelling, also reported that the arginine‐rich dipeptide, poly‐PR, was potently neurotoxic, whereas poly‐GR was less so 19. In this study, poly‐GA and poly‐GP peptides were without cytotoxic effect. Importantly, poly‐PR (and poly‐GR) proteins were observed to form nuclear, rather than cytoplasmic, aggregates and to strongly bind to nucleolar proteins, nucleoplasmin and fibrillarin, causing nucleoli to enlarge and triggering cell stress responses and death, whereas poly‐GA, poly‐GP and poly‐PA formed cytoplasmic aggregates 19. Consistent with this, Kwon and co‐workers 20 found that poly‐GR and poly‐PR proteins can enter cell nuclei, migrate to the nucleolus and poison RNA biogenesis. Hence, the present balance of experimental studies suggest that if DPR toxicity is indeed causal in human disease, then this could be mediated through the expression and accumulation of arginine‐rich dipeptides, poly‐PR in particular, which induce nucleolar stress.

Information on the neuronal distribution and specificity of the various DPRs in human brain, and spinal cord especially, is sparse. There is widespread brain presence of neuronal cytoplasmic inclusions (NCI) immunoreactive to poly‐GA, poly GP and poly‐GR, these apparently being similarly present in neuronal populations both vulnerable (frontal and temporal cortex) and nonvulnerable (occipital cortex, hippocampus and cerebellum) to FTLD pathology 10, 11, 12, 13, 14, 16, 21, 22. However, it should be pointed out that present studies attempting to map the topographic distribution of DPR in humans have mostly employed antibodies to poly‐GA 16, 21, 22, 23 or poly‐GR 13, 16, 23 proteins, and few have (also) included other DPR such as poly‐GP 16, 23 or poly‐PA 9, 23. Moreover, these have been essentially based on studies of the cerebrum, and have not usually investigated spinal cord. Classically, MND differs from FTLD by a lack of overt cognitive, behavioural and personality changes, and FTLD from MND by the absence of motor signs, although clearly there is overlap in the 15% of patients with FTLD that also display clinical MND 24, and the high proportion of patients with MND that can show cognitive changes 25. Therefore, it is important to examine spinal cord tissues, as a differential presence or absence of DPR in FTLD and MND within spinal neurones compared to cerebrum might be one of the features that underlie phenotypic variation.

In this study we have therefore examined sections from cerebral cortex, hippocampus, cerebellum, thalamus and spinal cord in 20 individuals with FTLD and/or MND bearing expansions in *C9orf72*, employing a panel of antibodies including all five DPR species produced through RAN translation. These regions were chosen as vulnerable (e.g. frontal and temporal cortex, spinal cord) and nonvulnerable (e.g. occipital cortex, hippocampus and cerebellum) regions, in MND at least. Cerebellar (Purkinje cells), thalamic and hippocampal CA3/4 neurones were also chosen as being large neurones, like anterior horn cells, with extensive arborization, it is possible that this might put them at greater risk *per se* than smaller granular neurones, such as those in dentate gyrus and cerebellum.

The principal aims of this study were thus two‐fold: to shed light on the expression and accumulation of DPR in different CNS regions by directly comparing all five DPR species in the same cell populations and on the same cases, and to ascertain whether spinal motor neurones might display differential patterns of DPR from cortical or cerebellar neurones which might explain their selective vulnerability in MND and inform pathogenesis.

## Methods

The study group consisted of 29 patients. The first group of 20 patients [five patients #1–5 with behavioural variant Frontotemporal dementia (bvFTD), six patients #6–11 with bvFTD combined with MND and nine patients #12–20 with MND alone] (Table S1) all bore an expansion in *C9orf72* (as evidenced by Southern blot and/or repeat primed PCR – see 11, 22) (Table S1). Four other individuals (patients' #21–24) had clinical MND, but did not carry an expansion in *C9orf72*. The remaining five individuals (patients' #25–29) were healthy controls not known to have suffered from motor or cognitive difficulties in life; none showed significant brain pathology. Fourteen of the 20 patients with expansions in *C9orf72* (patients #1–7, 9–11, 17–20) were from the North‐West of England and North Wales, and tissues were obtained from the Manchester Brain Bank through appropriate consenting procedures for the collection and use of the human brain tissues. The other six patients with expansions in *C9orf72* (patients #8 and 12–16) were obtained from London Neurodegenerative Diseases Brain Bank, as were the four MND cases without expansion in *C9orf72* (patients #21–24) and the five control cases (#25–29). Again, these were obtained through appropriate consenting procedures for the collection and use of the human brain tissues (reference number; Manchester Brain Bank, 09/H0906/52+5 for Newcastle and North Tyneside 1 REC; London Neurodegenerative Diseases Brain Bank, 08/MRE09/38+5 REC for Wales). The five patients with bvFTD (patients' #1–5) and those six with bvFTD+MND (patients' #6–11) fulfilled Lund‐Manchester clinical diagnostic criteria for FTLD 26, and were consistent with recent consensus criteria 27. All patients with MND (with or without bvFTD) fulfilled El Escorial criteria 28.

**Figure 1:**
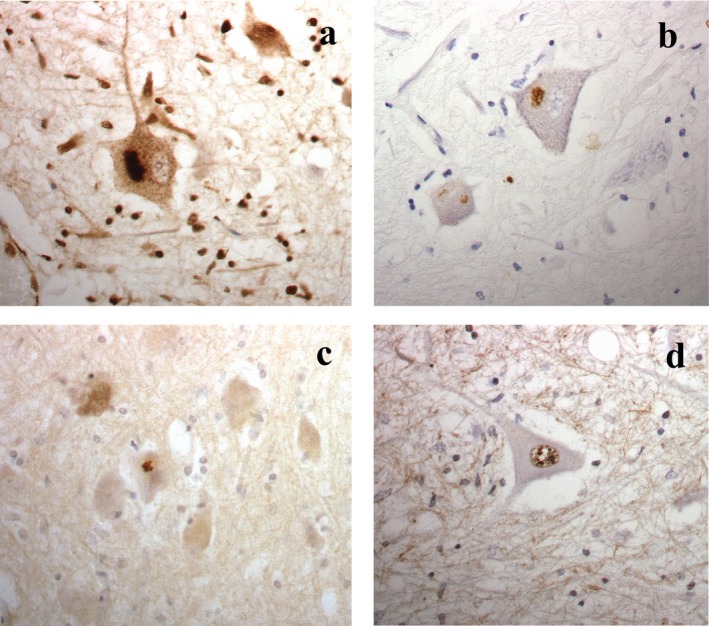
Surviving anterior horn cells in C9orf72 associated motor neurone disease show skein‐like or more rounded, solid neuronal cytoplasmic inclusions that are both TDP‐43 (**a**) and p62 (**b**) positive. Small, specular cytoplasmic inclusions immunopositive for poly‐GA protein are only rarely present (**c**). Neuronal nuclei are immunopositive for poly‐PR protein (**d**). Immunoperoxidase – haematoxylin × 40 microscope objective magnification.

Previous pathological diagnostic investigations had shown all five bvFTD + MND, and all 14 MND patients, to display atrophy and loss of motor neurones from trigeminal and hypoglossal cranial nerve nuclei and anterior horn cells (where spinal cord was available). Variable numbers of skein‐like, or rounded, more solid, TDP‐43 immunoreactive NCI, or fine, particulate accumulations of TDP‐43 were present in surviving cells, in which the nucleus had been ‘cleared’ of its normal physiological immunoreactivity (see Figure 1 **a**). No anterior horn cell loss was apparent in the spinal cord of the five healthy controls, and no TDP‐43 immunoreactive NCI were seen. Immunostaining with p62 antibody revealed a similar pattern of staining as TDP‐43, with skeins and more rounded, solid cytoplasmic inclusions being present (see Figure 1 **b**). The MND patients showed no extramotor TDP‐43 pathology, whereas all five patients with bvFTD+MND showed widespread TDP‐43 immunoreactive NCI within hippocampal dentate gyrus granule cells, and numerous cells in layer II of the frontal and temporal cortex contained TDP‐43 immunopositive granules with well‐formed NCI in others.

Seven antibodies raised against DPR were employed in this study. One set (Tokyo series of antibodies) included poly‐GA, poly‐GP and poly‐GR antibodies. These were raised against poly‐(GA)_8,_ poly‐(GP)_8_ and poly‐(GR)_8_ peptides with cysteine at N‐terminus, conjugated to *m*‐maleimidobenzoyl‐*N*‐hydrosuccinimide ester‐activated thyroglobulin. The thyroglobulin‐peptide complex (200 μg) emulsified in Freund's complete adjuvant was injected subcutaneously into a New Zealand White rabbit, followed by 4 weekly injections of peptide complex emulsified in Freund's incomplete adjuvant, starting after 2 weeks after the first immunization.

The second set of antibodies (Manchester series of antibodies) included poly‐GP, poly‐GR, poly‐PA and poly‐PR antibodies. These are rabbit polyclonal antibodies raised against the relevant putatively translated proteins. Briefly, peptides consisting of 15 GR, GP, PA or PR repeats with an additional N‐terminal cysteine were synthesized, and N‐terminally conjugated to keyhole limpet haemocyanin prior to immunization. Although initially custom‐made for our laboratory by Proteintech, the same antibodies are now commercially available (poly‐GR, 23978‐1‐AP; poly‐GP, 24494‐1‐AP; poly‐PA, 24492‐1‐AP; poly‐PR, 23979‐1‐AP). Details of these are provided by the manufacturers on their website (http://www.ptglab.com/Products/GA-repeat-Antibody-24492-1-AP.htm).

The specificity of both sets of antibodies for their native peptides was tested by ELISA, and by western blotting from cell lines expressing relevant DPRs (Data S1 and Figures S1 and S2). Following titration to determine optimal immunostaining, antibodies were employed at dilutions of 1:1000 (Tokyo poly‐GA, poly GP and poly‐GR), 1:750 (Manchester poly‐GR) and 1:500 (Manchester poly‐GP, poly‐PA and poly‐PR).

Both sets of antibodies were identically employed in a standard IHC protocol, as described previously 11, 22. Paraffin sections were cut at 6 μm from formalin fixed blocks of frontal, temporal and occipital cortex, hippocampus, thalamus, cerebellum and spinal cord (where available) from all 20 individuals bearing expansions in *C9orf72*. Samples of spinal cord and hippocampus alone were available from the four MND patients without an expansion in *C9orf72* (patients #21–24) and the five healthy controls (patients #25–29) (see Table S1). Where possible, sections of spinal cord were obtained from both cervical and lumbar enlargements, although in most instances only one of these regions (cervical) was available to study (Table S1). Further sets of sections were routinely immunostained 11 for p62 and TDP‐43 proteins, employing antibodies against non‐phosphorylated TDP‐43 (rabbit polyclonal antibody 10782‐2‐AP; Proteintech, Manchester, UK) and p62 protein (p62‐lck ligand, rabbit polyclonal antibody, BD Biosciences, Oxford, UK) at 1:3000 and 1:100 dilution, respectively, employing a standard ABC Elite kit (Vector, Burlingame, CA, USA) with DAB (3 3' diaminobenzidene) as chromagen. Antigen unmasking was performed in all immunoreactions by pressure cooking the sections in citrate buffer (pH 6.0, 10 mM) for 30 min, reaching 120°C and >15 kPa pressure. Stained sections were investigated microscopically for the presence of DPR, p62 and TDP‐43 immunoreactive inclusions.

The frequency of DPR immunostained inclusions within nerve cells was assessed blinded to diagnostic group (that is, bvFTD *vs*. bvFTD + MND *vs*. MND) by a single observer, similar to other published systems 23.

0 = no inclusions present in any field.

0.5 = rare/single inclusions in the entire section.

1 = few (<5) inclusions present, in some but not all fields.

2 = a moderate number (5–10) of inclusions present in each field.

3 = many (10–20) inclusions present, affecting most cells in each field.

4 = very many (>20) inclusions present, affecting nearly all cells in every field.

Reproducibility of the system was tested by repeat assessment of 10% of slides chosen at random, with agreement of the initial scoring being obtained in 97% of instances.

## Results

### Topographical comparisons of DPR immunoreactivity

#### Hippocampus and cerebellum

As we have reported previously 11, 22, TDP‐43 negative, p62‐positive NCI were widely present in the hippocampus and cerebellum of all individuals bearing an expansion in *C9orf72* irrespective of clinical or pathological diagnosis, but none were seen in these cells in any of those MND individuals without an expansion in *C9orf72*, nor in any of the controls. In dentate gyrus granule cells of the hippocampus, NCI appeared as small, rounded or grain‐shaped inclusions whereas in CA3/4 neurones these adopted a ‘star‐burst’ appearance. Similar, rounded or grain‐shaped, NCI were widespread within granule cells of the cerebellum, but inclusions with a ‘star‐burst’ appearance were only rarely seen in Purkinje cells, and in some individuals these were not present at all. DPR immunostaining showed NCI in granule cells of the dentate gyrus and cerebellum to be strongly reactive with poly‐GA and both poly‐GP antibodies, but on a case for case basis, fewer NCI were detected with each of the poly‐GR antibodies, even though the NCI themselves were still strongly immunostained. No NCI were detected with either poly‐PR or poly‐PA antibodies. The ‘star‐burst’ inclusions within CA3/4 neurones of hippocampus and Purkinje cells were strongly immunoreactive, as well as being numerically similar, with poly‐GA, both poly‐GP and both poly‐GR antibodies. Only rare inclusions within CA3/4 neurones (and none in Purkinje cells) were immunopositive with poly‐PA antibody. None of these cell types displayed any inclusions, either within the nucleus or cytoplasm, with either poly‐PR antibody, although as reported previously 11 there was some immunostaining of nuclei similar to that seen in anterior horn cells using poly‐PA and poly‐PR antibodies. There was no apparent loss of cells of any of these types from cerebellum or hippocampus, and all cells appeared healthy with no obvious changes to nuclear or nucleolar structure or size. No other neurodegenerative changes were observed.

#### Spinal cord

As expected, there was variable loss of anterior horn cells at all levels of the spinal cord (generally more severely so at cervical and thoracic, than lumbar, levels in all patients with bvFTD + MND and MND alone, although anterior horn cells appeared healthy in the single case of bvFTD alone (where the spinal cord was available for examination) and in the five heathy control subjects. TDP‐43 immunostaining showed variable numbers of immunoreactive NCI in surviving anterior horn cells in all except one (patient #1 with bvFTD) of the 11 cases bearing expansions in *C9orf72*, and in all four MND cases who did not carry an expansion in *C9orf72* (Figure 1 **a**). Clearing of normal physiological nuclear TDP‐43 staining was seen in neurones containing TDP‐43 immunoreactive NCI. No such NCI were seen in the spinal cord of the four healthy controls. Immunostaining with p62 antibody revealed a similar pattern of staining as TDP‐43, with skeins and more rounded, solid cytoplasmic inclusions being present (Figure 1 **b**).

Only very rare (usually only a single NCI per section) ‘star‐shaped’ poly‐GA or poly‐GP immunoreactive cytoplasmic, but not nuclear, inclusions were seen lying close to the cell surface of anterior horn cells in a few of the *C9orf72* expansion carriers (Figure 1 **c**). No immunoreactive inclusions were seen in either cytoplasm or nucleus in any case, with or without expansion in *C9orf72*, with poly‐GR or poly‐PA antibodies. None of the controls, or any of those individuals with MND but without an expansion in *C9orf72*, showed any DPR immunoreactive inclusions in the nucleus or cytoplasm of surviving anterior horn cells with any of the DPR antibodies. However, there was strong nuclear, but not cytoplasmic, staining of anterior horn cells in all cases, both in controls and in the MND cases with or without expansion in *C9orf72*, using the poly‐PR antibody (Figure 1 **d**). Even in otherwise normal appearing anterior horn cells, such immunostained nuclei had a ‘speckled’ appearance with larger clumps of immunopositive staining mixed in with a finer granular staining (Figure 1 **d**). In those cells clearly undergoing neurodegeneration, there was still heavy staining of a shrunken nucleus. The nucleolus remained unstained in all cells, and appeared to be of normal size in every region. Similar (to poly‐PR), although weaker, ‘speckled’ nuclear staining was seen with poly‐PA antibody in all cases.

### Comparisons of DPR staining by each antibody

Quantitative comparisons of frequency of DPR immunoreactive NCI were limited to measures obtained using poly‐GA, poly‐GR and poly‐GP antibodies, as antibodies to poly‐PA and poly‐PR were uninformative in this regard. All 20 patients bearing an expansion in *C9orf72* were assessed. Comparisons were made on frontal, temporal and occipital cortex, CA3/4 neurones of hippocampus, hippocampal dentate gyrus granule cells, granule cells of the cerebellum and neurones of the ventromedial thalamus. Comparisons were not made involving Purkinje cells of the cerebellum or anterior horn cells as the frequency of immunoreactive inclusions was too low to meaningfully compare.

**Figure 2:**
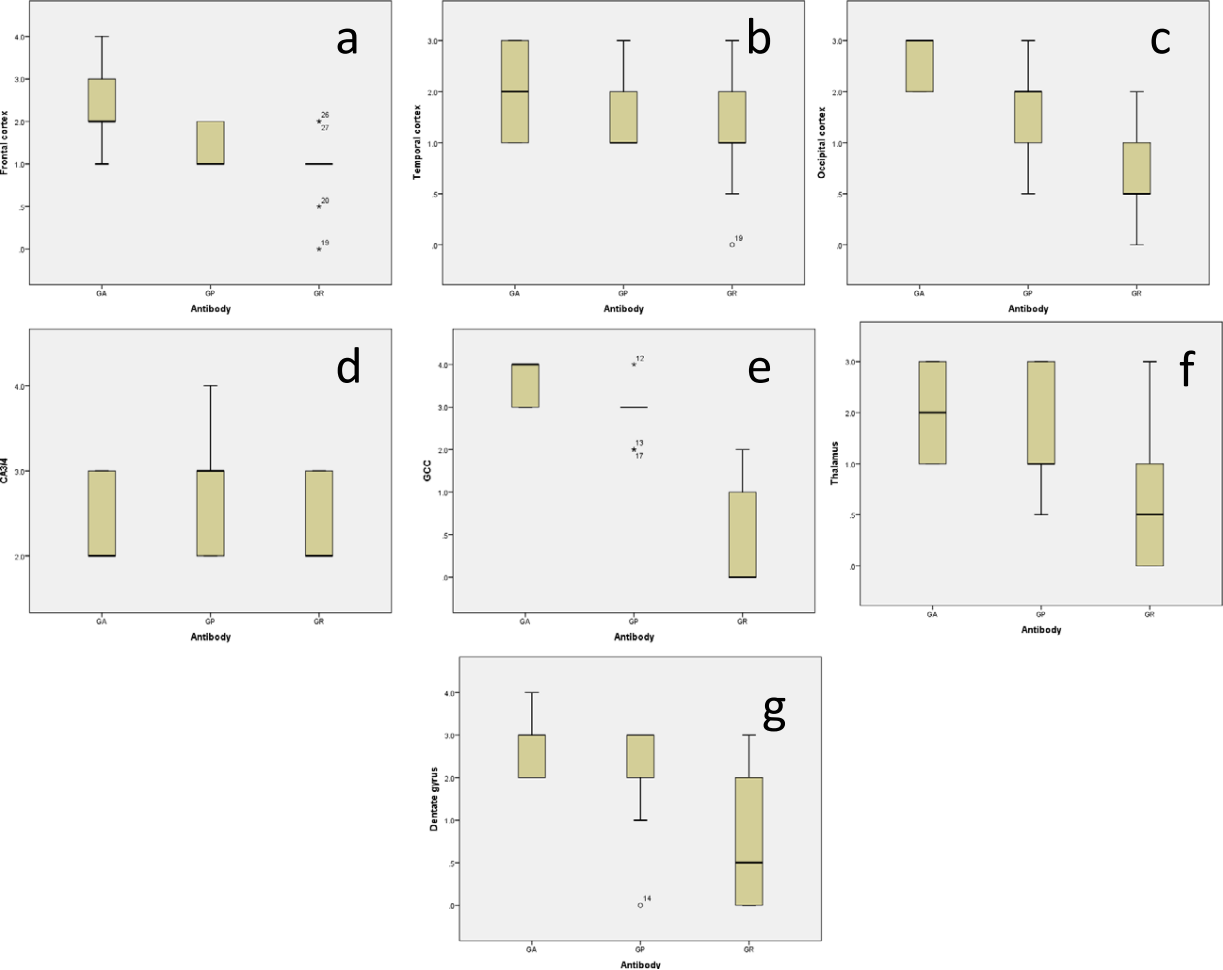
Boxplots for scores for dipeptide repeat proteins immunostaining employing Tokyo series of poly‐GA, poly‐GP and poly‐GR antibodies for frontal cortex (**a**), temporal cortex (**b**), occipital cortex (**c**), hippocampal CA4 pyramidal cells (**d**), granule cells of the cerebellum, GCC (E), thalamus (**f**) and dentate gyrus (**g**).

Comparisons by Kruskal–Wallis test showed that there were significant differences in DPR severity scores for DPR stained with the Tokyo series of antibodies between poly‐GA, poly‐GP and poly‐GR antibodies for frontal cortex (χ^2^ = 13.9, *P* = 0.001, Figure 2 **a**), occipital cortex (χ^2^ = 8.81, *P* = 0.012, Figure 2 **c**), cerebellar granule cells (χ^2^ = 13.2, *P* < 0.001, Figure 2 **e**), thalamus (χ^2^ = 20.3, *P* < 0.001, Figure 2 **f**) and but not for dentate gyrus (χ^2^ = 1.9, *P* = 0.381, Figure 2 **g**), temporal cortex (χ^2^ = 2.4, *P* = 0.308, Figure 2 **b**) or hippocampal CA3/4 pyramidal cells (χ^2^ = 1.6 *P* = 0.456, Figure 2 **d**). *Post hoc* testing (Mann–Whitney) (where Kruskal–Wallis test revealed significant differences in antibody scores) showed that scores for poly‐GA were not significantly different from those for poly‐GP in frontal (*P* = 0.698) and occipital (*P* = 0.277) cortex, thalamus (*P* = 0.369) and granule cells of the cerebellum (*P* = 0.165), but scores for both poly‐GA and poly‐GP were significantly greater than those for poly‐GR for all four regions (frontal cortex, *P* = 0.002 and 0.001, respectively; occipital cortex, *P* = 0.005 and 0.036, respectively; thalamus, *P* < 0.001 in both instances; cerebellum, *P* < 0.001 and *P* = 0.024, respectively) (Table S2).

Similarly, comparisons showed that there were also significant differences in DPR severity scores for DPR stained with the Tokyo poly‐GA antibody and the Manchester poly‐GP and poly‐GR of antibodies for frontal cortex (χ^2^ = 24.9, *P* < 0.001), temporal cortex (χ^2^ = 13.1, *P* = 0.001), occipital cortex (χ^2^ = 24.8, *P* < 0.001), dentate gyrus (χ^2^ = 20.7, *P* < 0.001), thalamus (χ^2^ = 25.4, *P* < 0.001) and cerebellar granule cells (χ^2^ = 34.8, *P* < 0.001), but not for hippocampal CA3/4 pyramidal cells (χ^2^ = 2.2 *P* = 0.328) (data not shown). *Post hoc* testing (Mann–Whitney) showed that scores for poly‐GA were significantly greater than scores for poly‐GP in frontal (*P* = 0.001) and occipital (*P* = 0.006) cortex and dentate gyrus (*P* = 0.014), marginally so for temporal cortex (*P* = 0.072), but not significantly different in cerebellum (*P* = 0.149) or thalamus (*P* = 0.602), and that scores for poly‐GA2 were significantly greater than those for poly‐GR for all regions (*P* < 0.001 in every instance). Scores for poly‐GP were also significantly greater than those for poly‐GR in all regions (frontal cortex, *P* = 0.021; temporal cortex, *P* = 0.038; occipital cortex, *P* = 0.006; dentate gyrus, *P* = 0.017; cerebellum and thalamus, *P* < 0.001 in both instances) (Table S2).

Conversely, there were no consistently significant differences across the various brain regions examined in scores for DPR stained with the Tokyo poly‐GP and Manchester poly‐GP antibodies (except for frontal cortex, *P* = 0.001), or with Tokyo poly‐GR and Manchester poly‐GR antibodies (except for temporal (*P* = 0.008) and occipital cortex (*P* = 0.005), dentate gyrus and cerebellum (both *P* = 0.001) where scores for Tokyo poly‐GR antibody were greater than those for Manchester poly‐GR antibody (Table S3). Likewise, comparisons of scores using the Tokyo antibodies between a clinically and pathologically affected (by TDP‐43) area of brain such as frontal cortex and a clinically ‘silent’ and pathologically uninvolved area such as occipital cortex showed that there were no significant differences in regional scores for DPR for poly‐GA (*P* = 0.546), poly‐GP (*P* = 0.436) or poly‐GR (*P* = 0.258) antibodies.

## Discussion

It is now well established that a widespread presence of NCI that are p62‐positive but TDP‐43‐negative, and which are composed of DPR, is a cardinal pathological feature of patients bearing hexanucleotide expansions in *C9orf72* 10, 11, 12, 13, 14. Indeed, such a pathological change is sufficiently robust as to provide a marker of the expansion, even in the absence of genetic analysis 11. In this study we have compared the extent of DPR inclusions in cerebral (frontal, temporal and occipital) cortex, hippocampus, thalamus and cerebellum, where they are most common 21, 22, in 11 cases of FTLD (five with bvFTD and six with bvFTD combined with MND) and 9 with MND, employing antibodies to poly‐GA, poly‐GP, poly‐GR, poly‐PA and poly‐PR. The present findings are consistent with our own previous findings 11, 22 and those of others 12, 13, 14, 16, 21, 23 (using the same 23 or different 12, 13, 14, 16, 21 antibodies as employed here) and show that, whereas the topographic distributions of DPR are remarkably similar for poly‐GA, poly‐GP and poly‐GR, in general numerically more inclusions are detected with poly‐GA than with poly‐GR, and in some regions, also with poly‐GP.

Dipeptide repeat proteins are formed through non‐ATG RAN translation which, in theory, implies that, with the exception of poly‐GP which is generated by both sense and antisense RAN translation, all DPR species should have an equal chance of being expressed. It is not reasonable to expect cells to express only a single peptide species, and given the close topographic similarities in expression pattern of poly‐GA, poly‐GP and poly‐GR containing aggregates, it is likely that these at least are always co‐translated. The strong correlations between scores for aggregates with poly‐GA, poly‐GP, poly‐GR and p62 antibodies 11 and the high degree of dual labelling of DPR with each antibody 16 emphasize this, and suggest that following translation, the individual dipeptides co‐aggregate into a single conglomerate. Nonetheless, preferential in‐frame read points might also influence the balance of peptide species produced. Indeed, there is some evidence of preferential sense‐transcription leading to higher expression of poly‐GA, poly‐GP and poly‐GR proteins over antisense poly‐PR and poly‐PA proteins 12, 29. Alternatively, sense products may be translated by other means, such as initiation from near‐ATG codons (e.g. CTG) 30, or from RAN translation initiated within the repeat region itself 14 through a self‐priming translation 31. Preferential formulation of such a structure facilitating poly‐GA/GP expression over poly‐GR expression could also help to explain the differential burden of DPR species we have observed in any given brain region.

Of course, it is possible that different antibody affinities for each DPR species could generate such variations. However, in this latter respect, we found here no difference in the pattern or extent of immunostaining between two independently raised sets of poly‐GR and poly‐GP antibodies implying antibody variation is not responsible. Differential aggregation or degradation tendencies could also play a role. The five DPR species have very different biophysical properties ranging from very hydrophobic poly‐GA to positively charged poly‐GR and poly‐PR peptides. Hence, poly‐GA, poly‐GP and poly‐PA proteins, being highly hydrophobic, would be predicted to be aggregation prone and more easily form inclusion bodies, whereas poly‐PR and poly‐GR, being positively charged, and thereby more soluble, may be less aggregation prone.

On the other hand, differential toxicities on the part of the DPR could also determine these different patterns of immunostaining for each DPR species. Experimental work suggests that the sense peptides, poly‐GA, poly‐GR and poly‐GP, and the antisense peptide poly‐PA, are not toxic, or at least have low toxicity 18, 19, although others have argued that poly‐GA does have toxic properties 15, 17. Conversely, DPR such as poly‐PR (and to a lesser extent, poly‐GR) have been shown in model systems to be potentially neurotoxic 18, 19. Poly‐PR and poly‐GR, being positively charged, and thereby more soluble and less aggregation prone, may be able to enter cell nuclei with an affinity for negatively charged molecules such as RNA, interfering with function 12, 14, 20. In this way, it has been hypothesized that cells expressing poly‐PR could be quickly poisoned and lost, and thereby no longer visible at end‐stage disease at *post mortem*. At present, it is possible that differences in expression pattern, combined with differential aggregation propensities, might determine the prevalence and relative peptide composition of individual inclusions seen at *post mortem* in bearers of expansion in *C9orf72*.

In the second part of this study, we investigated as to whether neuronal loss is associated with DPR formation and accumulation in order to gain insight as to whether differences in pattern of DPR immunostaining between spinal neurones and cerebral neurones might explain the selective loss of motor neurones in MND and bvFTD + MND compared to bvFTD alone. Present data showing widespread poly‐GA, poly‐GP and poly‐GR, but less prevalent poly‐PA, immunoreactive NCI within both vulnerable (frontal and temporal cortex) and non‐vulnerable (hippocampus and cerebellum) areas of the cerebrum, irrespective of whether overt cell loss was present or not, in cases with or without accompanying MND, are in keeping with previous reports 9, 11, 12, 13, 16, 21, 22, 23, 31. Such data are also consistent with experimental work 18, 19 showing that the sense peptides poly‐GA, poly‐GR and poly‐GP, and the antisense peptide poly‐PA, are not toxic, or at least have low toxicity. Collectively, present observations imply that these forms of DPR aggregates are likely to be accumulated within cells as ‘innocent bystanders’ without detriment to neuronal vitality or viability.

In contrast, we find here that sense direction, RAN‐translated, poly‐GA, poly‐GR, poly‐GP DPR are only rarely, if ever, seen in surviving motor neurones of the spinal cord. Such findings are consistent with other recent reports 23, 32, and collectively build on previous, but less extensive studies, based on poly‐GA 9, 12, 16, 21, 22, poly‐GP 16, poly‐GR 9, 12, 16, poly‐PA or poly‐PR antibodies 13, 16 antibodies. On the other hand, we did not detect any nuclear or cytoplasmic aggregates using poly‐PA or poly‐PR antibodies, but instead observed a strong nuclear staining of anterior horn cells in which the immunostained nuclei had a ‘speckled’ appearance with larger clumps of immunopositive staining mixed in with a finer granular staining by the coarser granules. Indeed, Mackenzie *et al*. 23 also remarked upon what seems to be the same kind of nuclear staining when using the antibody employed by Wen *et al*. 19. There are several reasons which might be responsible for these inconsistent findings. Clearly, differences in antibody specificity or avidity could be responsible. For example, Mori *et al*. 13 noted a lack of immunostaining with any DPR antibody in spinal neurones, although interestingly, and in contrast to other's workers observations, noted the star‐burst shaped inclusions in Purkinje cells and hippocampal CA3/4 neurones to be immunoreactive with poly‐PR antibody 12. Schludi and colleagues 16 noted occasional neuronal aggregates of poly‐PR within many brain regions, but interestingly none were seen in anterior horn cells in three cases of MND or bvFTD+MND. Others have also reported poly‐PR aggregates to be very rarely present in spinal cord 23, 32. On the other hand, Wen *et al*. 19 remarked on the presence of nuclear aggregates immunoreactive for poly‐PR in spinal cord sections although neither the cell type in which they were present, nor in how many cases they were seen was specified. Indeed similar poly‐PR immunoreactive aggregates were also seen, albeit to a much lesser extent, in nonexpansion bearers, and indeed in healthy controls.

Alternatively, methodological difference might explain such inconsistencies. This study and that of Mori *et al*. 13 employed a DAB‐based immunohistochemical protocol to detect aggregates/inclusions whereas Wen *et al*. 19 and Cooper‐Knock *et al*. 9 used immunofluorescence. Indeed, it is possible that the ‘aggregates’ detected by Wen *et al*. 19 and Cooper‐Knock *et al*. 9 (who incidentally employed the same poly‐PA and poly‐PR antibodies as used in this study) are represented by the ‘speckled’ nuclear staining we have described here. In agreement with Mackenzie *et al*. 23, we too would argue that these poly‐PR (and poly‐PA) ‘nuclear aggregates’ are unlikely to be disease‐associated for several reasons. Firstly, because we saw similar ‘aggregates’ to the same extent in anterior horn cells in non‐*C9orf72* MND, and even in healthy controls. Secondly, because both Purkinje cells of the cerebellum and CA3/4 neurones of the hippocampus, cell types not afflicted in MND, also showed similar a similar pattern of nuclear staining, Thirdly, the staining pattern seen resembled chromatin, and no nucleolar immunostaining or enlargement was seen, again in contrast to findings presented by Wen *et al*. who reported nucleolar enlargement as a prominent feature of cells expressing poly‐PR 19. Fourthly, we saw a similar, albeit weaker, pattern of nuclear staining in anterior horn cells, Purkinje cells and hippocampal CA3/4 neurones using poly‐PA antibody, yet poly‐PA has been demonstrated not to be neurotoxic 19. Taken together, these observations suggest that the poly‐PR antibody used here, and that employed in other reports 9, 19, might be cross‐reacting with some nuclear component (possibly bound to chromatin) with which it shares a repetitive PR sequence.

Alternatively, because experimental studies argue strongly for poly‐PR aggregates being highly neurotoxic 19, it could be argued that the lack of staining in surviving cells at *post mortem* might simply reflect the fact that those cells formerly containing/expressing such peptides had died and been lost from the tissue. However this might possibly be so in cervical regions of the spinal cord where cell death was always severe, disease‐specific poly‐PR aggregates might still have been anticipated to be present in lumbar regions of spinal cord where the degree of anterior horn cell preservation was better. The fact that none were seen argues against their invisibility being due simply to wipe out of affected cells prior to *post mortem*. Moreover, the reported presence of poly‐PR immunoreactive cytoplasmic inclusions in hippocampal and cerebellar neurones – cell types which are not lost in FTLD or MND – in patients with expansions in *C9orf72* 13 would further argue against poly‐PR neurotoxicity, or at least might suggest a resistance on the part of these cell types which is not shared by spinal motor neurones.

Conversely, and consistent with previous studies (see 33 for review), we and others 21, 23, 32 find that TDP‐43 containing inclusions are plentiful in surviving anterior horn cells of both expansion and nonexpansion bearers in MND and bvFTD + MND although, as might be expected, none were seen in bvFTD. TDP‐43 inclusions rarely co‐localize with DPR 21, 32. Collectively, these studies imply TDP‐43 mediated changes are much more likely than DPR to represent the pathological substrate of neurodegeneration in anterior horn cells. Such a viewpoint is emphasized by present observations that although DPR were common in cells of CA3/4 region of hippocampus, no loss of neurones was observed, and no TDP‐43 immunoreactive NCI were present.

In summary, present findings and those of others 23, 32, based on observations of human brains, would argue that even though formation of DPR can clearly induce neurotoxicity in model systems, RAN translation of the expansion leading to formation and aggregation of DPR does not seem to be a factor which determines anterior horn cell death in *C9orf72* associated disease. The marked discrepancies between the patterns of DPR aggregates in nuclei and cytoplasm between model systems and observations in humans also advise caution. The rate of production of DPR is likely to be very low in patients, especially during early life, with these abnormal proteins accumulating gradually over decades. In contrast, transient overexpression of DPR, particularly in cultured cells, will quickly overwhelm many protein degradation systems, such as the proteasome and autophagy pathways, leading to cytoplasmic and nuclear aggregations, and inducing a cascade of catastrophic cellular events including nucleolar fragmentation and stress, ultimately resulting in cell death. Therefore, such experimental phenotypes must be interpreted conservatively. Nevertheless, in parallel to Alzheimer's disease or Parkinson's disease, where soluble forms of amyloid β protein or α‐synuclein are considered to be the toxic species rather than the large aggregated masses of these proteins within plaques or Lewy bodies, it remains possible that soluble monomeric or oligomeric forms of DPR, especially poly‐PR, could, themselves, interfere with TDP‐43 function and RNA metabolism, and in so doing ultimately trigger neurodegeneration. However, recent studies 23 indicating that such soluble species cannot be detected in brain tissue from human expansion carriers would further argue against a potential role for either soluble or insoluble DPR species in the pathogenetic mechanism in C9orf72 associated disease.

## Author contributions

DM devised and designed the study, performed all microscopical assessments, data analysis and wrote the paper. YD, XL and DW performed the immunohistochemistry. AR prepared sections from the Manchester cases for immunostaining and helped with statistical advice and data analysis. CT prepared sections for immunostaining, and provided clinical data, from the Institute of Psychiatry cases. SR and SP‐B performed the genetic analyses. JS, JS and JT helped with preparation of the manuscript. MJ, AR, HH, JE and JS characterized, and provided clinical data for, the Manchester cases. MM‐S, GS, TN and MH prepared the Tokyo DPR antibodies, and characterized both Manchester and Tokyo DPR antibodies by ELISA and western blotting.

## Conflict of interest

All authors declare no conflict of interest.

## Supporting information


**Figure S1.** Specificity of antibodies for their antigenic protein as determined by ELISA, for anti GR (A), anti GP (B) anti AP and anti PR (both C) antibodies.

**Figure S2.** Specificity of antibodies for their antigenic protein as determined by Western blotting of proteins extracted from cell lines expressing the relevant peptide.

**Table S1.** selected clinical, neuropathological and genetic data on the 29 cases employed in the study.

**Table S2.** *Post hoc* (Mann–Whitney) significance values for comparisons between scores for DPR immunostaining using antibodies against poly‐GA, poly‐GP and poly‐GR proteins following attainment of significant difference when comparing scores for all three antibodies by Kruskal–Wallis test.
**Table S3.** Significance values for comparisons of scores (by Mann–Whitney test) for DPR immunostaining in different brain regions using Manchester and Tokyo poly‐GP, and Manchester and Tokyo poly‐GR antibodies.
