# Supplementary material for: Neurodegeneration in frontotemporal lobar degeneration and motor neurone disease associated with expansions in C9orf72 is linked to TDP‐43 pathology and not associated with aggregated forms of dipeptide repeat proteins
Source: Neuropathol Appl Neurobiol. 2015 Dec 7;42(3):242–54. doi: 10.1111/nan.12292 (PMC4832296; doi:10.1111/nan.12292)
Supplement: Supplementary file 1 — Figure S1. Specificity of antibodies for their antigenic protein as determined by ELISA, for anti GR (A), anti GP (B) anti AP and anti PR (both C) antibodies. [file NAN-42-242-s001.docx]

**A**

**B**

**C**

Supplementary Figure 1
