# Supplementary material for: Neurodegeneration in frontotemporal lobar degeneration and motor neurone disease associated with expansions in C9orf72 is linked to TDP‐43 pathology and not associated with aggregated forms of dipeptide repeat proteins
Source: Neuropathol Appl Neurobiol. 2015 Dec 7;42(3):242–54. doi: 10.1111/nan.12292 (PMC4832296; doi:10.1111/nan.12292)
Supplement: Supplementary file 2 — Figure S2. Specificity of antibodies for their antigenic protein as determined by Western blotting of proteins extracted from cell lines expressing the relevant peptide. [file NAN-42-242-s002.docx]

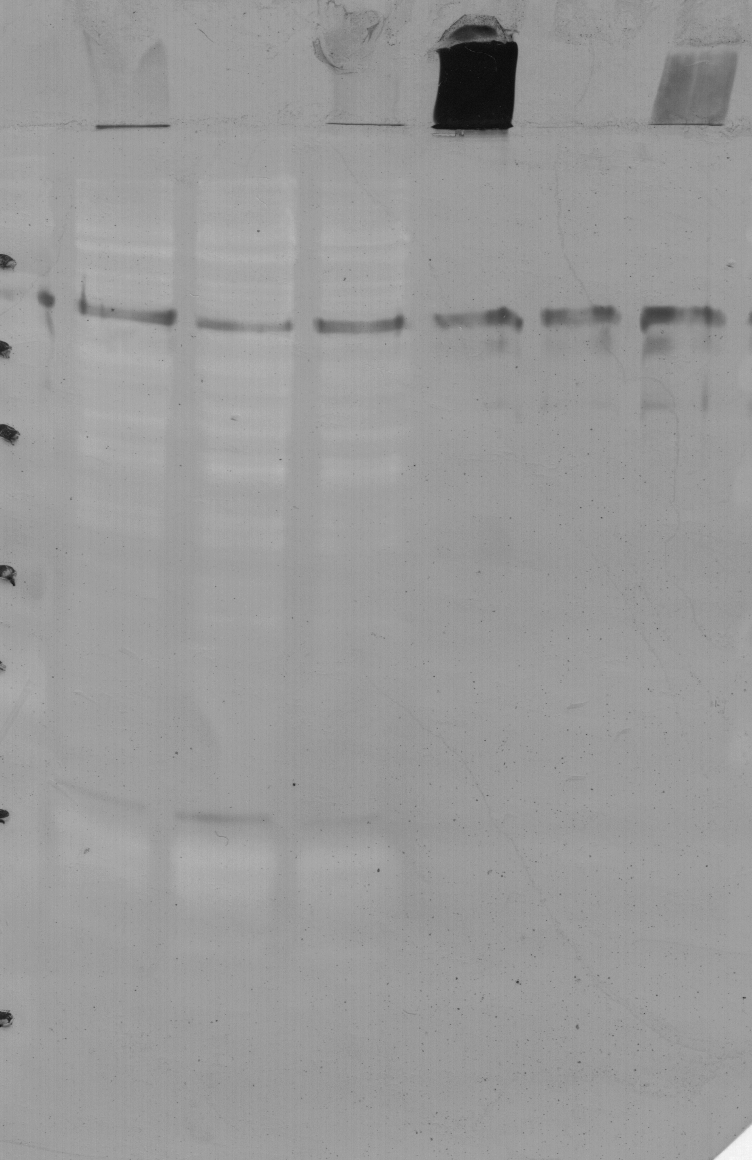

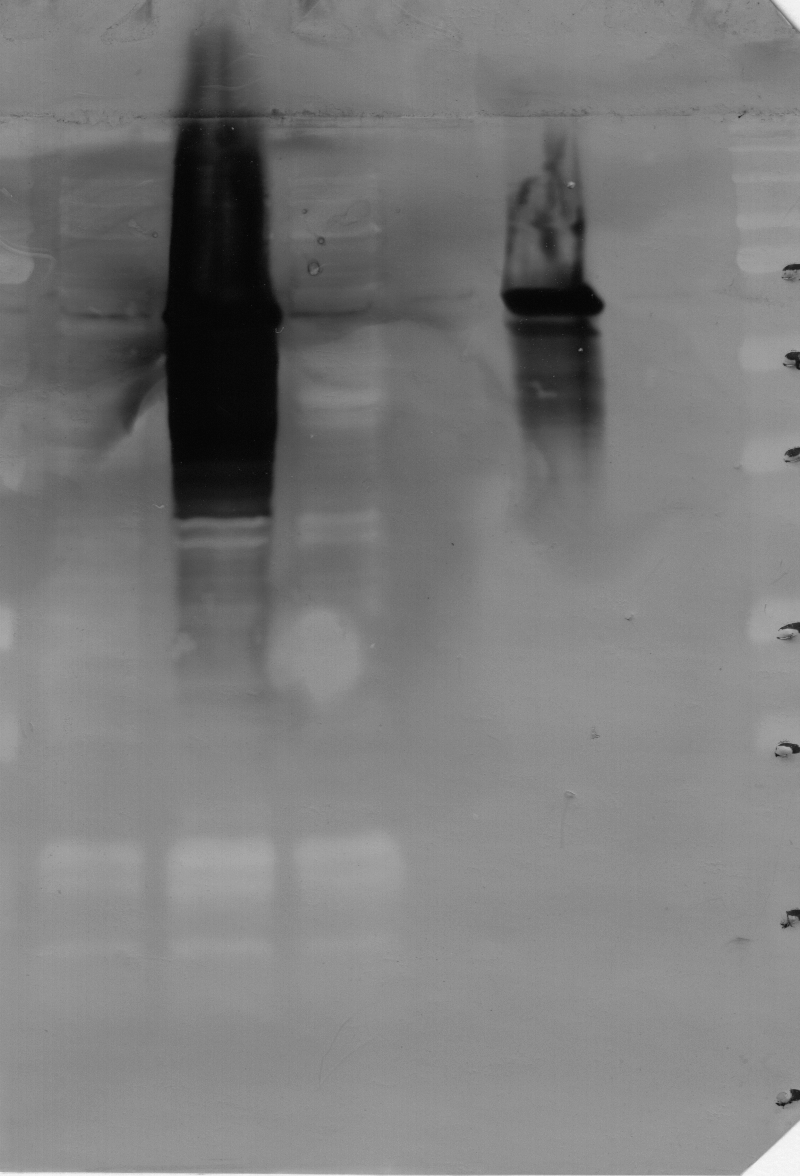

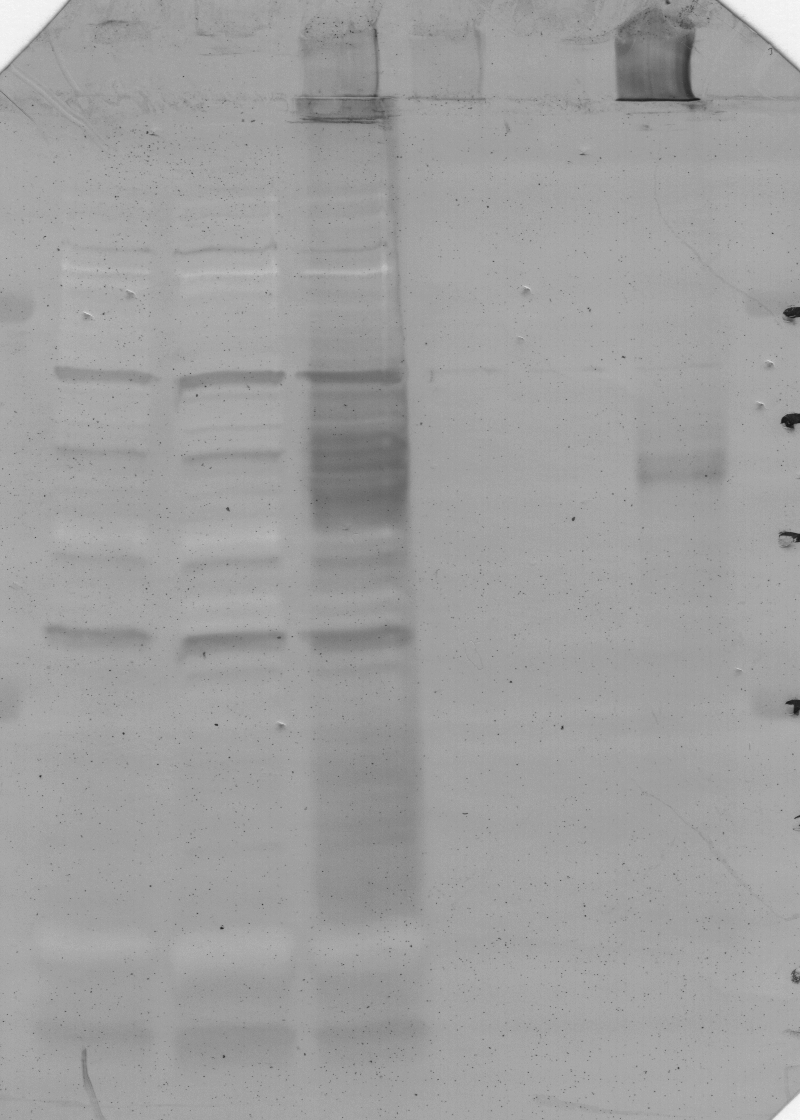

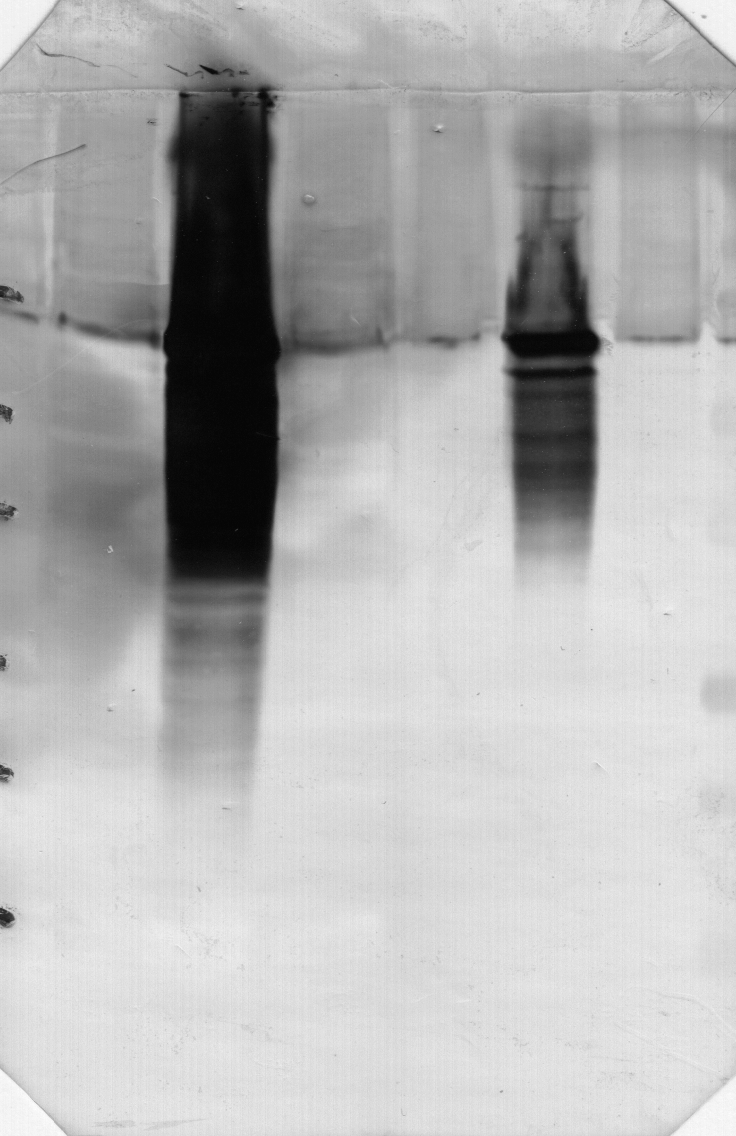

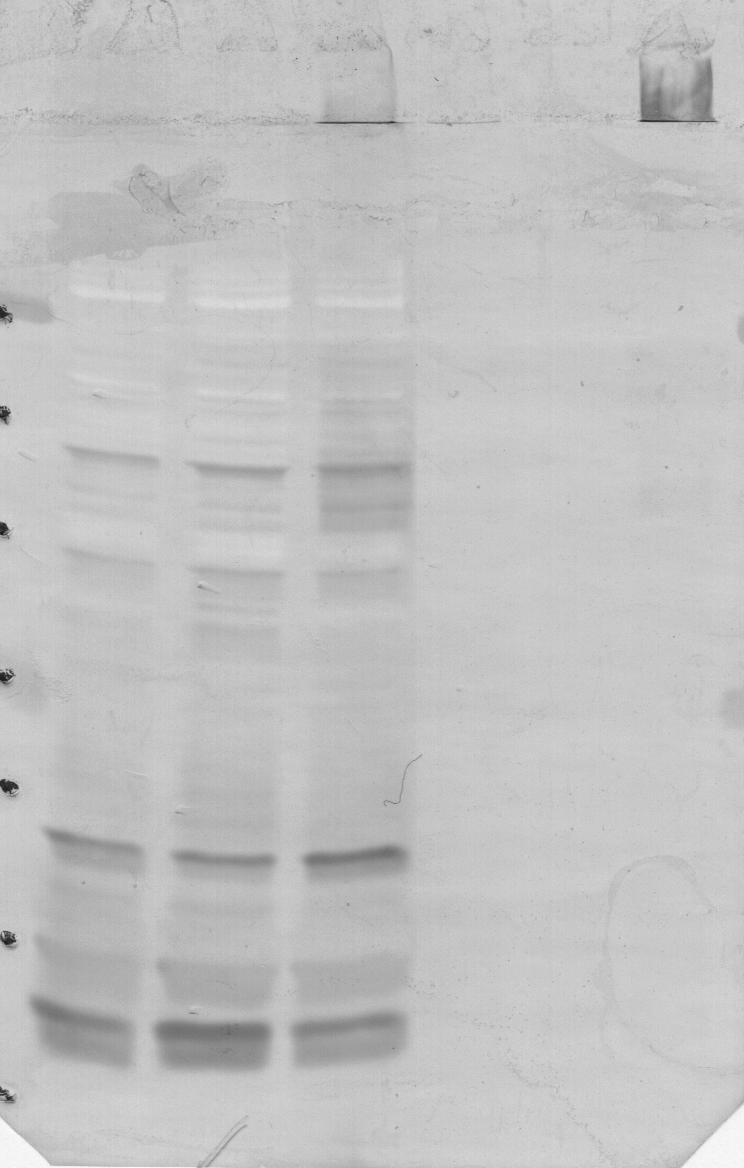


20

25

37

50

75

10

15

20

25

37

50

75

10

15

stacking gel

1

2

3

Sar-sup

Sar-ppt

1

2

3

1

2

3

Sar-sup

Sar-ppt

1

2

3

1

2

3

Sar-sup

Sar-ppt

1

2

3

1

2

3

Sar-sup

Sar-ppt

1

2

3

1

2

3

Sar-sup

Sar-ppt

1

2

3

stacking gel

anti-GA-2

anti-Manchester

anti-GP Manchester

anti-GP-1

anti-GR-1

1: GFP-poly GA

2: GFP-poly GP

3: GFP-poly GR
