# Supplementary material for: Neurodegeneration in frontotemporal lobar degeneration and motor neurone disease associated with expansions in C9orf72 is linked to TDP‐43 pathology and not associated with aggregated forms of dipeptide repeat proteins
Source: Neuropathol Appl Neurobiol. 2015 Dec 7;42(3):242–54. doi: 10.1111/nan.12292 (PMC4832296; doi:10.1111/nan.12292)
Supplement: Supplementary file 4 — Table S2. Post hoc (Mann–Whitney) significance values for comparisons between scores for DPR immunostaining using antibodies against poly‐GA, poly‐GP and poly‐GR proteins following attainment of significant difference when comparing scores for all three antibodies by Kruskal–Wallis test. Table S3. Significance values for comparisons of scores (by Mann–Whitney test) for DPR immunostaining in different brain regions using Manchester and Tokyo poly‐GP, and Manchester and Tokyo poly‐GR antibodies. [file NAN-42-242-s004.docx]

Supplementary Table 2

|  |  | Poly-GA | Poly-GP | Poly-GR |
| --- | --- | --- | --- | --- |
| Frontal Cortex | Poly-GA |  | 0.001 | 0.001 |
|  | Poly-GP | 0.698 |  | 0.021 |
|  | Poly-GR | 0.002 | 0.001 |  |
|  |  |  |  |  |
| Temporal Cortex | Poly-GA |  | 0.072 | 0.001 |
|  | Poly-GP | NS |  | 0.038 |
|  | Poly-GR | NS | NS |  |
|  |  |  |  |  |
| Occipital Cortex | Poly-GA |  | 0.006 | 0.001 |
|  | Poly-GP | 0.277 |  | 0.006 |
|  | Poly-GR | 0.005 | 0.086 |  |
|  |  |  |  |  |
| Hippocampus CA3/4 | Poly-GA |  | NS | NS |
|  | Poly-GP | NS |  | NS |
|  | Poly-GR | NS | NS |  |
|  |  |  |  |  |
| Cerebellar Granule Cells | Poly-GA |  | 0.149 | 0.001 |
|  | Poly-GP | 0.165 |  | 0.001 |
|  | Poly-GR | 0.001 | 0.024 |  |
|  |  |  |  |  |
| Thalamus | Poly-GA |  | 0.602 | 0.001 |
|  | Poly-GP | 0.369 |  | 0.001 |
|  | Poly-GR | 0.001 | 0.001 |  |
|  |  |  |  |  |
| Dentate Gyrus | Poly-GA |  | 0.014 | 0.001 |
|  | Poly-GP | NS |  | 0.017 |
|  | Poly-GR | NS | NS |  |

Supplementary Table 2: Post-hoc (Mann-Whitney) significance values for comparisons between scores for DPR immunostaining using antibodies against poly-GA, poly-GP and poly-GR proteins following attainment of significant difference when comparing scores for all 3 antibodies by Kruskal-Wallis test. Figures in black relate to comparisons between Tokyo series of antibodies, figures in red relate to comparisons involving Tokyo poly-GA antibody and Manchester poly-GP and poly-GR antibodies. NS indicates no significant differences between scores for all 3 antibodies by Kruskal-Wallis test.

|  | Tokyo poly-GP  vs Manchester poly-GP | Tokyo poly-GR  vs Manchester poly-GR |
| --- | --- | --- |
| Frontal Cortex | 0.001 | 0.149 |
| Temporal Cortex | 0.026 | 0.008 |
| Occipital Cortex | 0.102 | 0.005 |
| Hippocampus CA3/4 | 0.678 | 0.989 |
| Cerebellar Granule Cells | 0.968 | 0.001 |
| Thalamus | 0.640 | 0.081 |
| Dentate Gyrus | 0.142 | 0.001 |

Supplementary Table 3

Supplementary Table 3: Significance values for comparisons of scores (by Mann-Whitney test) for DPR immunostaining in different brain regions using Manchester and Tokyo poly-GP, and Manchester and Tokyo poly-GR antibodies.
