# Supplementary material for: Neurodegeneration in frontotemporal lobar degeneration and motor neurone disease associated with expansions in C9orf72 is linked to TDP‐43 pathology and not associated with aggregated forms of dipeptide repeat proteins
Source: Neuropathol Appl Neurobiol. 2015 Dec 7;42(3):242–54. doi: 10.1111/nan.12292 (PMC4832296; doi:10.1111/nan.12292)
Supplement: Supplementary file 5 [file NAN-42-242-s005.docx]

ELISA

Immunoreactivity of all antisera was characterized by ELISA as follows. The peptide immunogens were coated onto microtiter plates. The plates were blocked with 10 % fetal bovine serum (FBS) in PBS, incubated with the rabbit antisera diluted in 10 % FBS/ PBS at room temperature for 1.5 h, followed by incubation with HRP-goat anti-rabbit IgG (Bio-Rad) at 1:3000 dilution, and reacted with the substrate, 0.4 mg/mL *o*-phenylenediamine, in citrate phosphate buffer (24 mM citric acid, 51 mM Na_2_HPO_4_). The absorbance at 490 nm was measured using Plate Chameleon (HIDEX). Antibodies were used at optimal dilutions of 1:1000-1:3000.

Using both Manchester and Tokyo antibodies and immunising peptides, ELISA demonstrated that both sets of antibodies had similar properties (Supplementary Figure 1). Both Manchester (UK) poly-GR and Tokyo (J) poly-GR antibodies reacted with GR15 (Manchester (UK)) and GR8 (Tokyo (J)) peptides similarly and specifically (A). Both Manchester (UK) poly-GP and Tokyo (J) poly-GP1 antibodies reacted with GR15 (Manchester (UK)) and GR8 (Tokyo (J)) peptides similarly and specifically, but both antibodies immunoreacted with GP15 (Manchester) peptide more strongly than GR8 (Tokyo) peptide (B). Manchester poly-AP and poly-PR antibodies reacted with Manchester AP15 and PR15 peptides, respectively, and specifically (C).

Western Blotting

The specificity of the antibodies was tested by western blotting of DPR isolated from cell lines expressing relevant constructs. cDNAs encoding 100 repeats of poly-GA, poly-GP or poly-GR without a GGGGCC repeat [28] were synthesized (Life Technologies) and subcloned into pEGFP-C1 (Clontech). SH-SY5Y cells were transiently transfected with each plasmid (1 µg) using XtreamGENE9 (Roche). After 2-days incubation, cells were harvested and lysed in 300 µL of homogenization buffer (10 mM Tris-HCl, pH 7.5 containing 0.8 M NaCl, 1 mM ethyleneglycol bis-aminoethyl ether-*N,N,N,N*-tetra acetic acid (EGTA), 1 mM DTT and 1% N-Lauroyl sarcosine sodium salt (Sarkosyl) by brief sonication. The lysates were centrifuged at 100,000 g for 20 min at room temperature. The supernatant was recovered as Sarkosyl (Sar)-soluble fraction (Sar-sup). The pellet was suspended in 100 µL SDS-sample buffer and sonicated. The resulting samples were used as the Sar-insoluble fraction (Sar-ppt). Each sample was separated by SDS-PAGE (12% gel) and immunoblotted with the indicated antibodies. Both Tokyo and Manchester sets of antibodies were used.

Using Tokyo anti-GA antibody, the GFP-poly-GA protein (predicted size, ~42 kDa) was mainly detected within the stacking gels in Sar-ppt fraction, while no bands were observed in Sar-sup, indicating that the GFP-poly-GA protein is prone to aggregation and form a high-molecular-weight complex (Supplementary Figure 2). This antibody also weakly recognized the GFP-poly-GR protein (predicted size, ~45 kDa) within the stacking gel in Sar-ppt. The GFP-poly GP protein (predicted size, ~45 kDa) was observed with Tokyo anti-GP antibody, as unexpected bands of MW approximately between 30~100 kDa, suggesting peculiar SDS-binding properties and/or folding because of the decreased electromobility of proteins containing highly repeated sequences (Supplementary Figure 2). This antibody barely reacted with the other GFP-DPR. Using Tokyo anti-GR antibody, we observed not only ~45 kDa band, but also high-molecular-weight complex of GFP-poly GR protein in Sar-sup and ppt, suggesting that GFP-poly GR protein is also aggregation-prone. This antibody slightly reacted with GFP-poly GA protein within the stacking gel in Sar-ppt (Supplementary Figure 2). A similar result was obtained for Manchester poly-GP and poly-GR antibodies (Supplementary Figure 2).
